# Supplementary figures and images for: A phenome-wide association study of 26 mendelian genes reveals phenotypic expressivity of common and rare variants within the general population
Source: PLoS Genet. 2020 Nov 23;16(11):e1008802. doi: 10.1371/journal.pgen.1008802 (PMC7735621; doi:10.1371/journal.pgen.1008802)

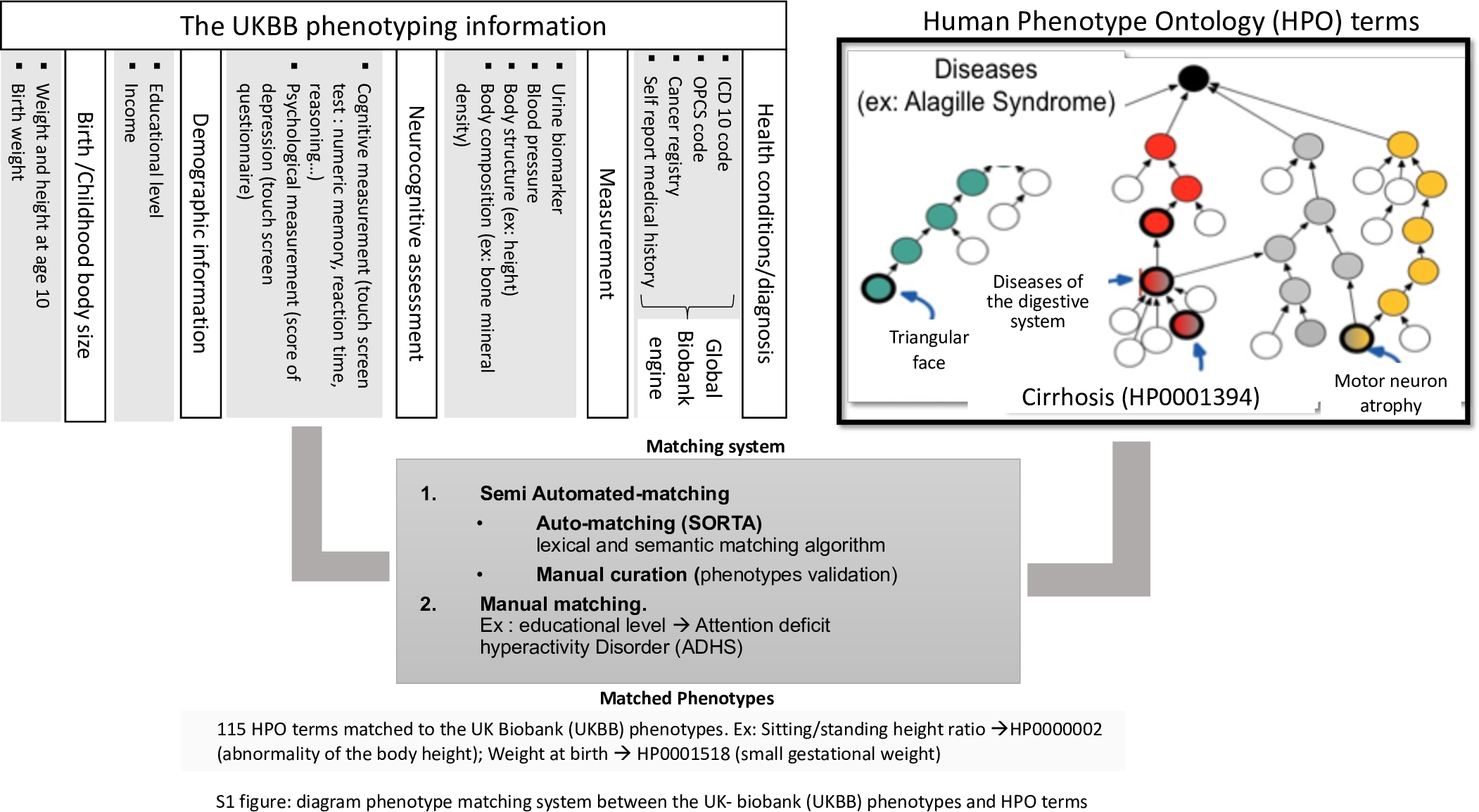

Supplement: S1 Fig — (TIF) [file pgen.1008802.s010.tif]

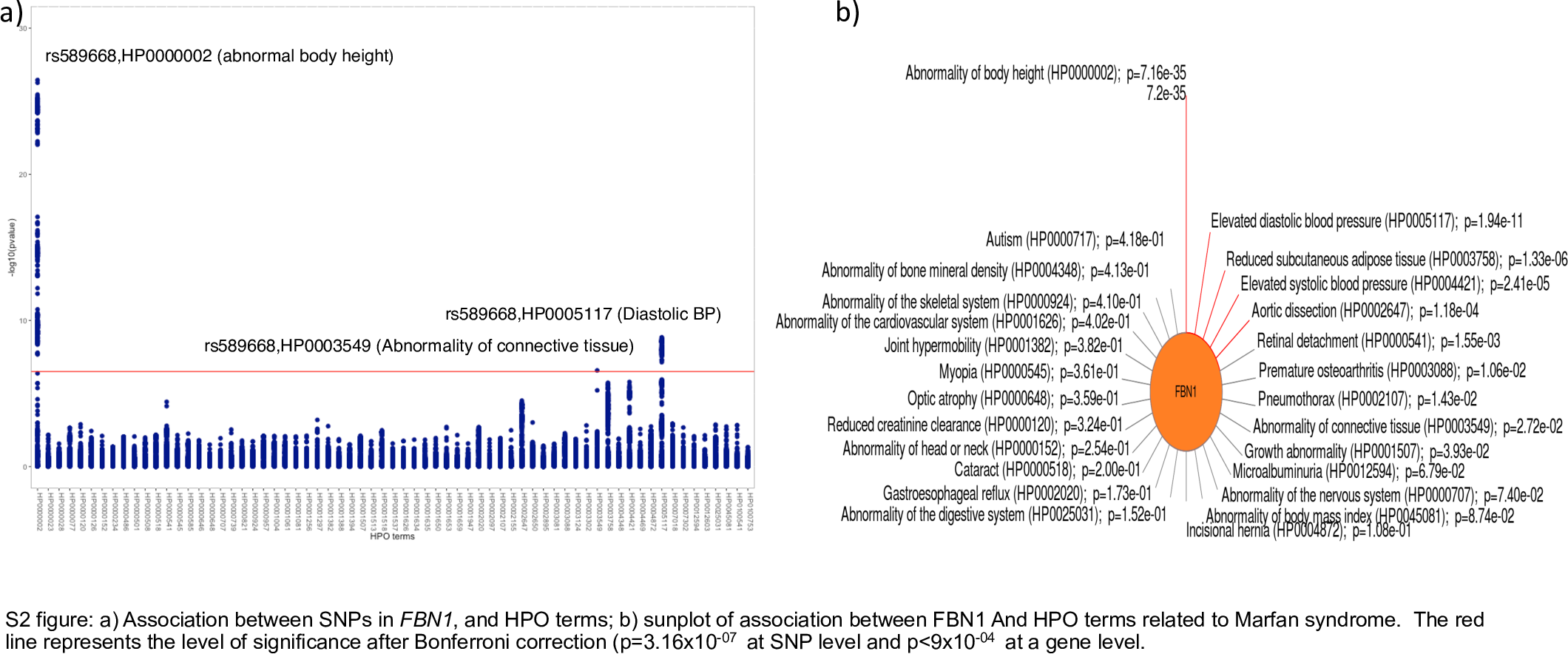

Supplement: S2 Fig — (TIF) [file pgen.1008802.s011.tif]

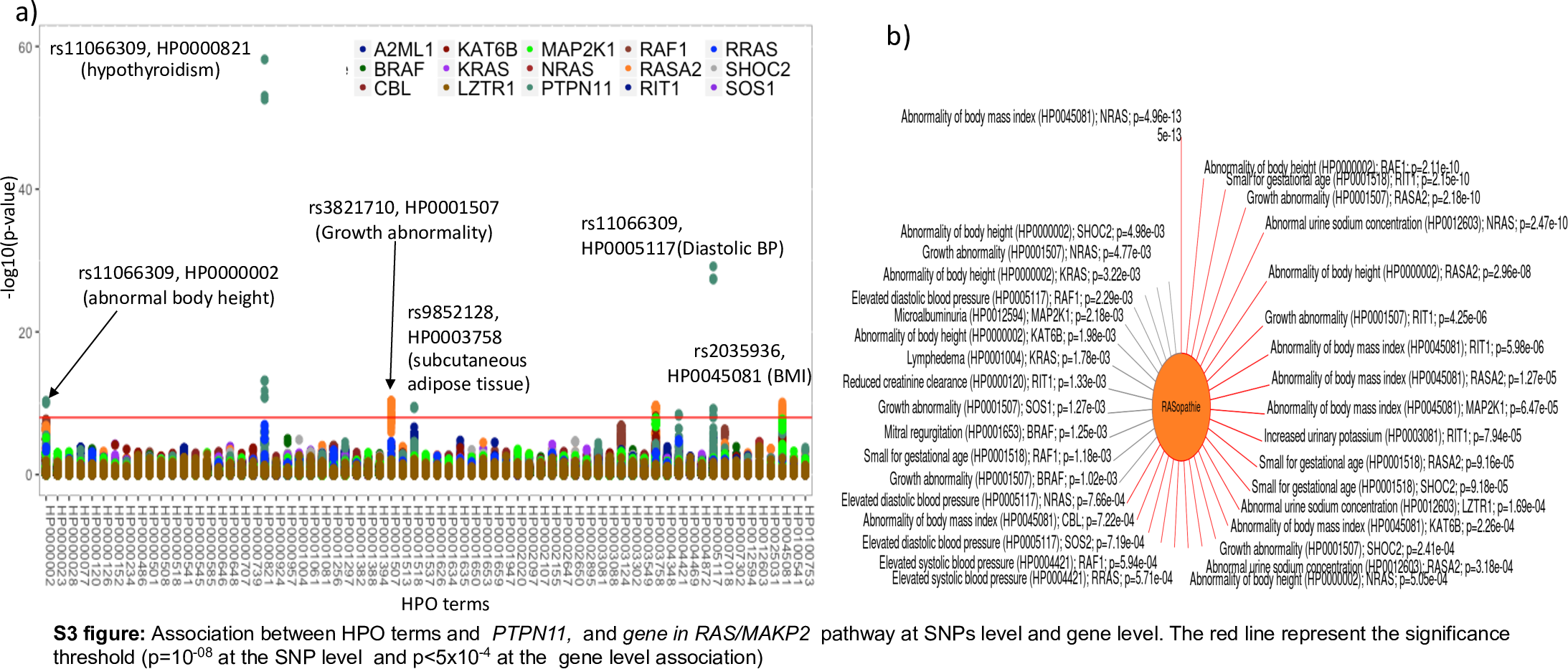

Supplement: S3 Fig — (TIF) [file pgen.1008802.s012.tif]

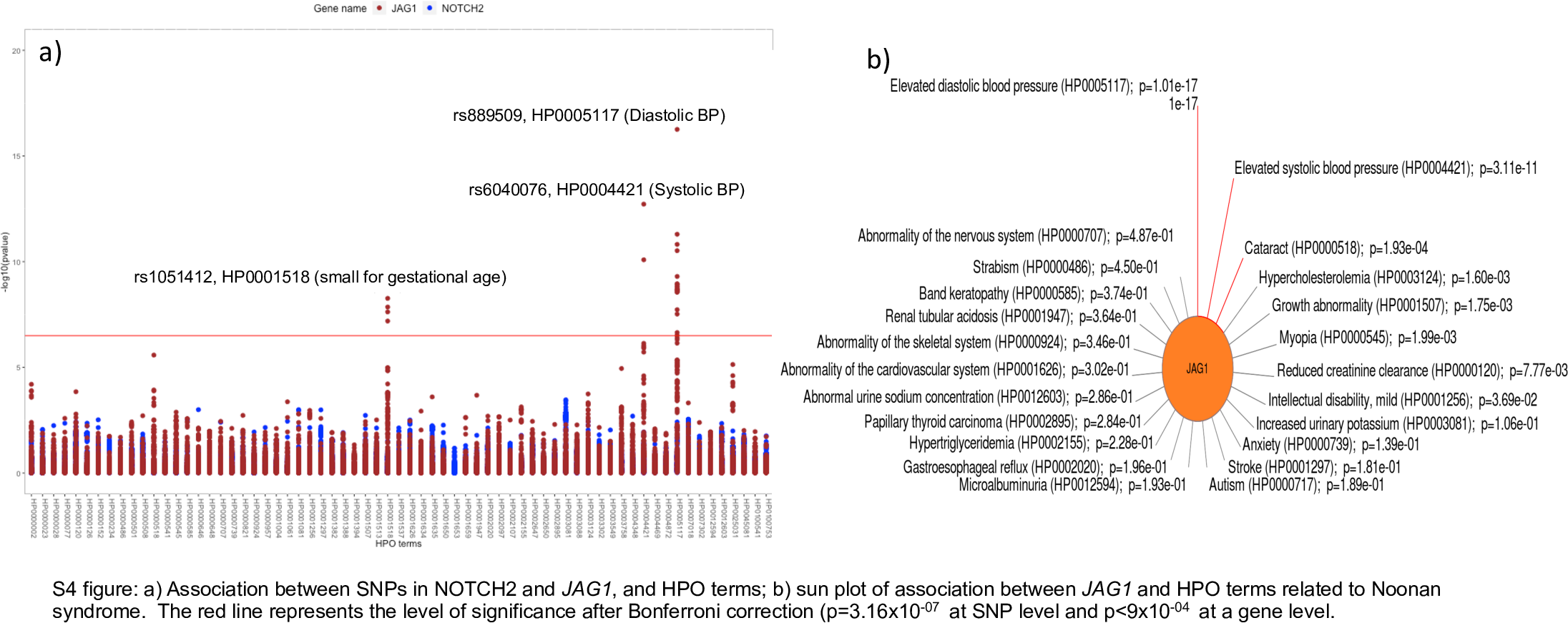

Supplement: S4 Fig — (TIF) [file pgen.1008802.s013.tif]

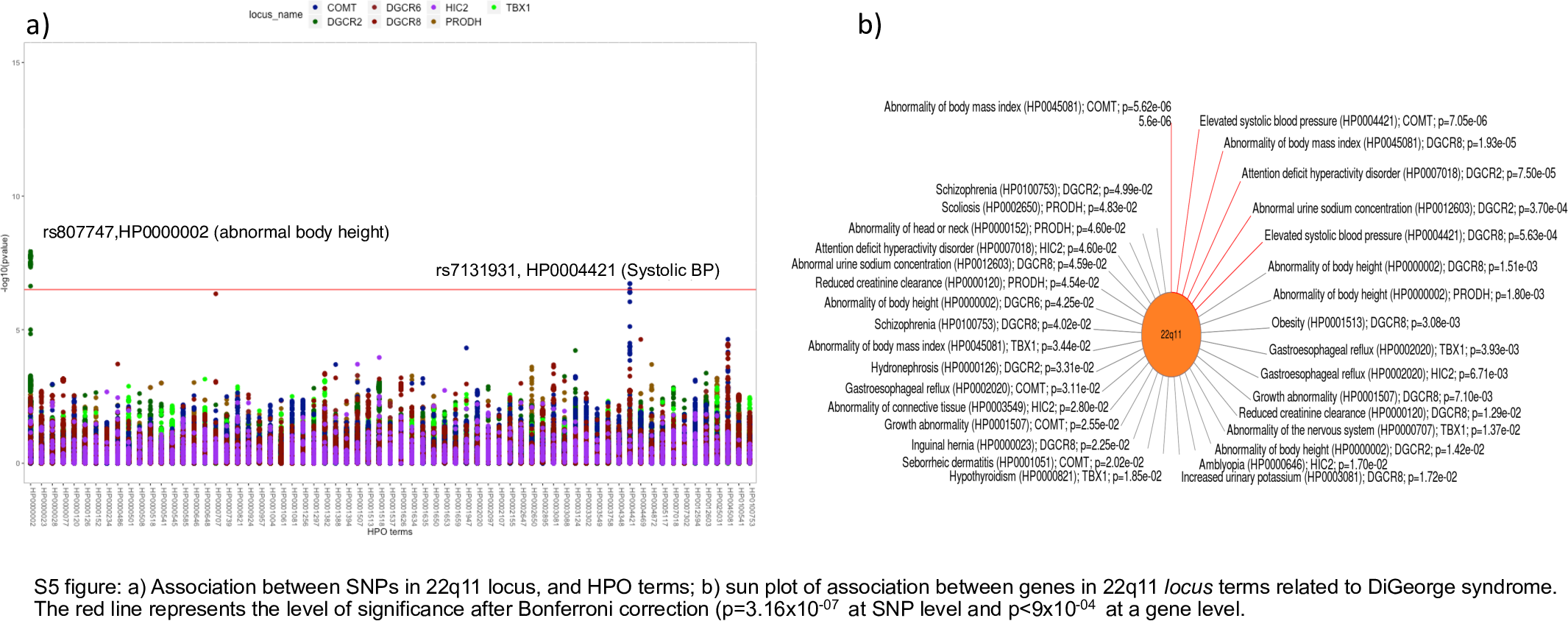

Supplement: S5 Fig — (TIF) [file pgen.1008802.s014.tif]

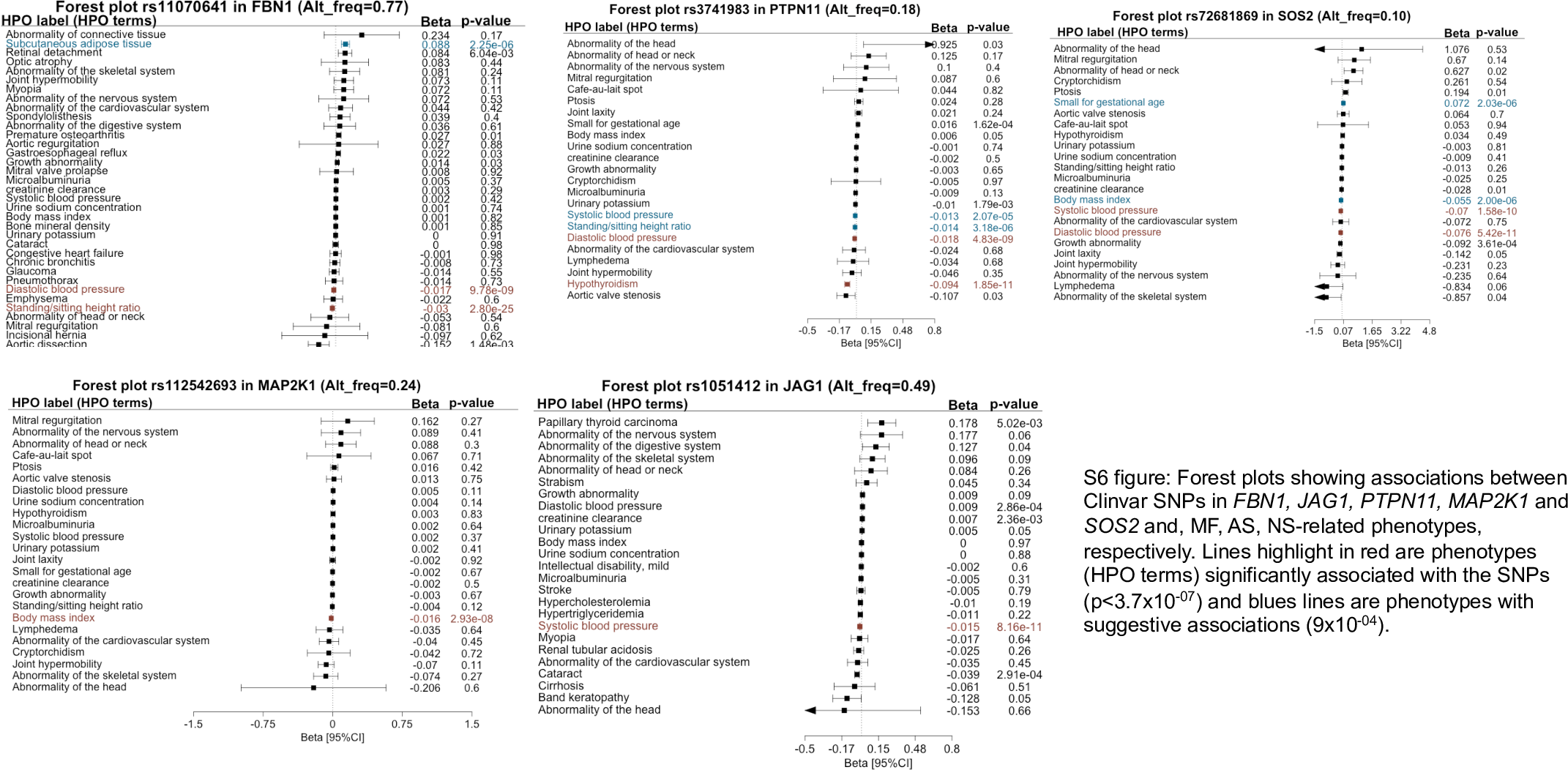

Supplement: S6 Fig — (TIF) [file pgen.1008802.s015.tif]

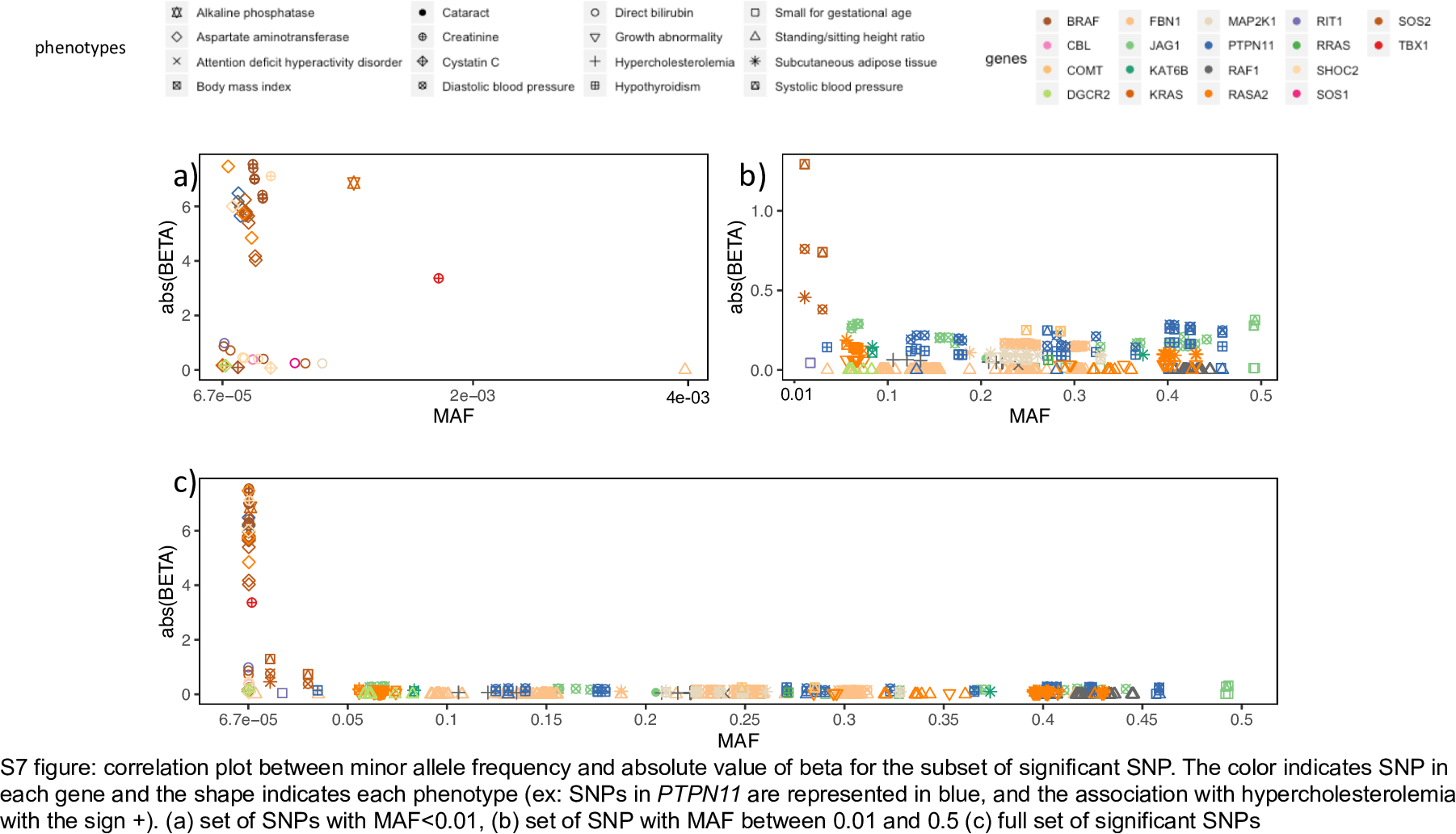

Supplement: S7 Fig — The color indicates variant in each gene and the shape indicates each phenotype (ex: variants in PTPN11 are represented in blue, and the association with hypercholesterolemia with the sign +). (a) set of variants with MAF<0.01, (b) set of variant with MAF between 0.01 and 0.5 (c) full set of significant variants. (TIF) [file pgen.1008802.s016.tif]
