# Supplementary material for: A phenome-wide association study of 26 mendelian genes reveals phenotypic expressivity of common and rare variants within the general population
Source: PLoS Genet. 2020 Nov 23;16(11):e1008802. doi: 10.1371/journal.pgen.1008802 (PMC7735621; doi:10.1371/journal.pgen.1008802)
Supplement: Attachment — Submitted filename: 20191113_ReviewComments_submitted.docx [file pgen.1008802.s017.docx]

This article puts forth an analysis of UKBB data exploring the role of common and rare variants in genes that cause rare mendelian (monogenic) disorders, Alagielle Syndome (AS), Marfan Syndrome (MS), DiGeorge Syndrome (DS), and Noonan Syndrome (NS). Together, variants in 26 genes make up these syndromes and the authors then identified a list of HPO-derived phenotypes for each syndrome to identify component phenotypes. These phenotypes were matched in UKBBB to identify a list of phenotypes that each SNP would be tested against to determine if there was a significant association, a PheWAS study. PheWAS was performed at the per-SNP and the per-Gene level using SKAT.

The authors identified several SNPs and genes that are associated with discrete categorial or quantitative phenotypes in the UKBBB. This study links SNPs located within genes associated with Mendelian syndromic disorders with a variety of component phenotypes. These findings are quite interesting to the genetics community, as bridging our understanding of how common and rare variants can influence clinical traits and phenotypes are much needed within the community. While the effects of identified SNPs are relatively small and a lone would not be expected to have a significant clinical impact if found in the general population, these s study do demonstrate that even for non-Mendelian variants, variants in these genes can influence expression of phenotype -matched components of the gene.

My major concerns with the study are in the design and presentation of the manuscript. While the title of the article highlights identification of pleiotropy, starting with syndromic disease genes that are inherently pleiotropic is problematic. The abstract however, does not mention pleiotropy and this seems to be less important throughout the manuscript perhaps a rewording of the title and manuscript might provide a more accurate contextualization of the interesting findings here.

Overall, the manuscript would benefit from definitions upfront for what would be considered rare vs common variant MAF, as. In addition, there is some confusing wording that makes reading the manuscript challenging because specific terminology has typically been used for rare disease vs complex trait genetics, and therefore depending on the background of the reader certain assumptions are made. I outlined some suggestions for improving clarity below, where I could, but my suggestions are by no means comprehensive and I may have misinterpreted what one was trying to say.

**Major**

- My major comment is over the idea that one is identifying novel pleiotropy through pheWAS of phenotypes related to genes where pathogenic mutations cause known *syndromic* genetic disorders seems a little circular. Monogenic genetic syndromes, in many ways are prime examples of pleiotropy, where a single gene affects many different and often unrelated developmental phenotypes. As such syndromic genetic disorders are pleiotropic.
- I suspect that m any of the differences here are related to our definition of the word “pleiotropy”. While this work demonstrates that many genetic variants in LD are demonstrating pleiotropy, pleiotropy definitions I have seen refer to a gene, rather than variants/alleles within the gene. If demonstrating novel pleiotropy is one of the intents of the manuscript, it would be more convincing if they looked at a larger number of phenotypes, those related to the syndromes and then other randomly selected phenotypes representing the other organ systems that are not known to be part of the clinical syndrome.
- Some key question that remains unanswered in my mind, are: is the OR or Beta higher when looking at more rare SNPs? Is the OR or Beta more significant because it is in an established monogenic syndrome gene? i.e. should we be weighting variants that are known to have significant effect sizes in the rare-disease space as compared to variants that are in non-monogenic disease genes? And, importantly, might these effects be clinically significant for diagnosis? Some of these might be interesting to address in the discussion or supplemental materials.
- The major limitation here is in the phenotype data, which the authors acknowledge in the discussion. Many of the “component” phenotypes of the syndromes are not observed in the UKBBB cohort, which are primarily adults and therefore limited in pediatric phenotypes, particularly those due to dysmorphic features. Is there an independent validation cohort biobank that you could perform your analysis in, such that you could directly test these SNPs to look at specific traits (i.e TSH levels or stroke)?
- How were SNPs selected, i.e. are they all within the gene boundary or did you also look some distance 5’ to TSS and 3’ to the termination codon? Please add this description to your methods. Have you explored additional methods to pick the SNPs regulating genes? It might be more informative to pick SNPs that are eQTLS for the gene in question, or ones that are known to be in contact through 3D confirmation?
- Does allele frequency seem to influence to OR or Beta value of the trait? Does it influence the p-value. Do the number of SNPs tested per gene influence performance of the gene-based SKAT test?
- Line 225 states “While classical Mendelian forms of each syndrome may be uncommon, individual phenotypes attributable to these syndromic loci appear to be common within the general population”, this is a little bit confusing, as the phenotypes are influenced by variants in the loci but they are not “attributable” to these variants. I might change the statement to read: … “individual phenotypes that *are modulated by variants within* the syndromic loci appear to be appear to be *present* within the general population.”. I think the effect you are seeing is more similar to identifying a risk locus for complex trait (individual phenotype)
- In the discussion (line 228), the point is made that their results support the use of a continuous phenotyping score to improve identification of rare disease using EHR. I don’t believe that referenced study seeks to identify individuals with rare syndromic mendelian disease that are using phecodes (component phenotypes) present in the EHR. I do think that that referenced paper demonstrates presence of a specific pattern component disease phenotypes might indicated presence of pathogenic Medelian genetic mutations. However, it does not really talk about how common variants in the same gene can effect matched individual phenotypes to the syndrome.
- Can you corroborate other findings for significant SNPs/Loci that overlap with previous GWAS or PheWAS significant loci? I see the eQTL data mentioned in the manuscript, but well-phenotyped GWAS studies have been done on many of the anthromophoric phenotypes used here. While you may not have a causal variant in hand, maybe you have one of the causal genes that is being regulated.
- Line 86 mentions that syndromic disease is due to rare and familial alleles, while the following paragraph talks about “common and rare alleles” to explore pleiotropy. Its not clear how one distinguishes rare, pathogenic vs rare and affecting individual component. How would you know that the individual does not have syndromic features—it is possible that some of the individual patients with “rare” alleles could be mildly affected (i.e. undiagnosed genetic disease?)

**Minor:**

1. How many are common, how many are rare variants that you are looking at? What is your MAF definition of rare variant vs common variant? Recent work in Wright, CF et all 2019, showed that genotyping of rare variants was notoriously inaccurate, so it would be good to know the lower limit of MAF you are considering.
2. For SNPS associated with Height or BMI—have they also been implicated in large scale GWAS studies?
3. Figure 1: Please incorporate information that distinguishes Figure 1a from 1b in the legend. It is not immediately clear what the difference is, it appears that 1a is SNP-based tests that is Bonferroni corrected (and therefore corrected for multiple testing) and 1b is due to gene-based testing by SKAT.
4. In the main text, line130, it says “After correction for multiple testing”, and at first pass suggested that data in Figure 1A was not corrected for multiple testing. After reading several times, I realize that correction was performed, however this would benefit from simply flipping the sentence structure so you introduce gene based testing first and then mentioned that “after correction for…. Had significant hits….”
5. Last sentence of Abstract: This sentence is a little unclear to me. I might flip the sentence to say: rare and common variants within established syndromic disease genes can have an effect on the syndrome’s individual component phenotypes within the general population.
