# Supplementary material for: A phenome-wide association study of 26 mendelian genes reveals phenotypic expressivity of common and rare variants within the general population
Source: PLoS Genet. 2020 Nov 23;16(11):e1008802. doi: 10.1371/journal.pgen.1008802 (PMC7735621; doi:10.1371/journal.pgen.1008802)
Supplement: Attachment — Submitted filename: Rebuttal letter PheWAS_final02152020.docx [file pgen.1008802.s018.docx]

**Response to reviewers**

*On behalf of all the authors Dr. Tcheandjieu and Dr. Priest wish to offer our sincere thanks to all three reviewers for taking the time to engage with our science and provide thoughtful criticism. We sincerely believe that the work is improved due to the timely and constructive comments from all three reviewers.*

**Reviewer #1**: The authors present a study on common polymorphisms linked to Mendelian disease genes and show that some GWAS hits are associated with phenotypic features associated with the Mendelian diseases. This finding is not entirely new, although the authors do not cite much of the previous literature, e.g., Translating Mendelian and complex inheritance of Alzheimer's disease genes for predicting unique personal genome variants (PMC3277633), The Human Phenotype Ontology: Semantic Unification of Common and Rare Disease (PMC4572507), amd Phenotype-Specific Enrichment of Mendelian Disorder Genes near GWAS Regions across 62 Complex Traits (PMID:30290150), and others.

One novelty in the current study is related to its use of UK Biobank data, and the phenotypic focus of the current work, which I believe goes beyond what I am familiar with in the literature.

*We thank the reviewer for their comments and suggestions on previous literature--we have added these citations to the manuscript and clarified the introduction. We have added the following sentence in the introduction :*

*Recent studies have shown that rare and common variants in or near mendelian diseases genes are associated with complex traits in the general population[9–11]. Moreover, Freund M.K. et al[11], have demonstrated an enrichment of signal from Genome Wide Association Studies (GWAS) near syndromic disease genes for the related phenotypes. However, this work was based on the curation of available GWAS summary statistics.*

1. There is a major mistake in the way the authors are using ClinVar data. They state:

"Four additional SNPs (rs11070641, rs4775760, rs363832 and rs140605) that reach genome-wide significance with high standing/sitting height ratio and diastolic BP were correlated with several syndromic disease entities in CLINVAR including stiff skin syndrome, ectopia lentis, MASS syndrome, thoracic aortic aneurysm and aortic dissection (SupplementaryTable 3). However, rs11070641 (which is <https://www.ncbi.nlm.nih.gov/clinvar/variation/316310/>) is listed as ClinVar as benign. The Table at ClinVar is listing conditions that in principle can be tested for FBN1 mutations (including say stiff skin syndrome). However, it is not saying that the variant is linked with these diseases! Stiff skin syndrome is caused by highly specific mutations in exon 37 of FBN1 that affect an integrin binding site (review the OMIM entry <https://omim.org/entry/184900> for details). rs11070641 is a 3UTR variant.

The authors should review all associations that have been derived in this way. It would be useful to have the actual evidence for association with disease reflect the actual data and not a ClinVar assertion (which is second hand so to speak, and the data are often noisy as in this case).

*We thank the reviewer for their comments and appreciate the suggestion that the way we have presented the CLINVAR data is confusing. We have now moved this information to the discussion with the goal of using CLINVAR classifications as “context”, noting that mutations in CLINVAR which lack evidence for causality for mendelian disease, may nonetheless contribute to the risk for specific phenotypes or act as modifiers for syndromic disease when a “pathogenic’ variant is present. The modified sentence in the discussion is as follows:*

*Four additional SNPs (rs11070641, rs4775760, rs363832 and rs140605) in FBN1 were associated with high standing/sitting height ratio, a characteristic feature often observed in Marfan syndrome. These variants are reported as benign variants in CLINVAR suggesting that they do not cause primary syndromic disease, but our data suggest they may be modifiers of penetrance.*

2. SNPs in PTPN11 display a moderate to low correlation with each other suggesting several independent signals within the locus => Do the authors have a biological explanation for the observation that only the PTPN11 gene was predicted to have independent signals?

*We thank the reviewer for their comments, we have modified the sentence in the results with the following:*

*SNPs in PTPN11 display a moderate to low correlation, while high correlation was observed between SNPs in RASA2, SOS2 and MAP2K1 indicating that the association observed within each gene represents a single signal (Figure 4b). To identify independent signals within each gene for each associated phenotype, we performed conditional regression using stepwise selection procedure implemented in GCTA. For each subset of associated SNPS-phenotypes pairs within each gene, we identify one independent signal (Figure 1b).*

*After performing conditional analysis, for the association between SNPS in PTPN11 and the set of associated phenotypes, we identified 3 independent signals in the regions. However, we do not have a clear biological explanation on this particular observation. We acknowledge as mentioned in the discussion (paragraph 4 line 330 - 339), that, the observed association with PTPN11 can be driven by genes located upstream or downstream PTPN11.*

3. Modifiers of penetrance and phenotypic expressivity in Marfan syndrome have been proposed,[15,16] but our results suggest that common variants and local haplotype structure around syndromic genes may deserve more attention[17]. => This has been discussed by several papers in the literature including Hutchinson S, Furger A, Halliday D, Judge DP, Jefferson A, Dietz HC, Firth H, Handford PA. Allelic variation in normal human FBN1 expression in a family with Marfan syndrome: a potential modifier of phenotype? Hum Mol Genet. 2003 Sep 15;12(18):2269-76. Epub 2003 Jul 22. PubMed PMID: 12915484.

*We thank the reviewer for their comments. The reference has been add and discussed in the following sentence:*

*A study based on a single Marfan syndrome family also suggested that differences in normal FBN1 expression could contribute to the clinical variability of Marfan syndrome phenotype[19].*

Minor

1. The Y axis in Figure 1 is nearly impossible to read without magnification. The figure should be redone so that it can be interpreted at the size of a normal journal figure.

*We thank the reviewer for the comment. Figure has been updated and the text font on the Y axis increased.*

I do not follow what the authors mean by "HPO terms are represented by shape"? Please add a legend!

*We thank the reviewer for the comment. In the figure, we used different shapes to represent the association between SNPs in each gene and different phenotypes. We have corrected the sentence in the figure title with the following:*

The color indicates SNP in each gene and the shape indicates each phenotype (ex: SNPs in *PTPN11* are represented in blue, and the association with diastolic blood pressure indicated with the sign +).

2. line 175 typo: lower level of cutaneous adipocytes tissues => lower level of cutaneous adipocyte** tissues

*We thank the reviewer for their comments and have corrected the text accordingly.*

3. Figure 3 legend typo: RAS-opathie genes => RAS-opathy genes

*We thank the reviewer for their comments and have corrected the text accordingly.*

4. in FBN1 rs589668 (MAF=0.25 in Europeans populations) => in FBN1 rs589668 (MAF=0.25 in European** populations)

*We thank the reviewer for their comments and have corrected the text accordingly.*

5. line 239 typo: GTex => The correct acronym is GTEx

*We thank the reviewer for their comments and have corrected the text accordingly.*

**Reviewer #2:** This article puts forth an analysis of UKBB data exploring the role of common and rare variants in genes that cause rare mendelian (monogenic) disorders, Alagille Syndrome (AS), Marfan Syndrome (MS), DiGeorge Syndrome (DS), and Noonan Syndrome (NS). Together, variants in 26 genes make up these syndromes and the authors then identified a list of HPO-derived phenotypes for each syndrome to identify component phenotypes. These phenotypes were matched in UKB to identify a list of phenotypes that each SNP would be tested against to determine if there was a significant association, a PheWAS study. PheWAS was performed at the per-SNP and the per-Gene level using SKAT.

The authors identified several SNPs and genes that are associated with discrete categorical or quantitative phenotypes in the UKB. This study links SNPs located within genes associated with Mendelian syndromic disorders with a variety of component phenotypes. These findings are quite interesting to the genetics community, as bridging our understanding of how common and rare variants can influence clinical traits and phenotypes are much needed within the community. While the effects of identified SNPs are relatively small and a lone would not be expected to have a significant clinical impact if found in the general population, these study do demonstrate that even for non-Mendelian variants, variants in these genes can influence expression of phenotype -matched components of the gene.

*We thank the reviewer for their positive comments.*

My major concerns with the study are in the design and presentation of the manuscript. While the title of the article highlights identification of pleiotropy, starting with syndromic disease genes that are inherently pleiotropic is problematic. The abstract however, does not mention pleiotropy and this seems to be less important throughout the manuscript perhaps a rewording of the title and manuscript might provide a more accurate contextualization of the interesting findings here.

*We thank the reviewer for their incisive analysis here and below in detail and upon reflection we agree that perhaps pleiotropy is not quite the right concept. Instead our analyses are really getting more at the idea of expressivity of individual traits from a pleiotropic loci (defined by clear syndromic associations). We have changed the title of the manuscript to,* ***“A phenome-wide association study of four syndromic genes reveals phenotypic expressivity of common and rare variants within the general population”***

Overall, the manuscript would benefit from definitions upfront for what would be considered rare vs common variant MAF, as. In addition, there is some confusing wording that makes reading the manuscript challenging because specific terminology has typically been used for rare disease vs complex trait genetics, and therefore depending on the background of the reader certain assumptions are made. I outlined some suggestions for improving clarity below, where I could, but my suggestions are by no means comprehensive and I may have misinterpreted what one was trying to say.

**Major**

-	My major comment is over the idea that one is identifying novel pleiotropy through pheWAS of phenotypes related to genes where pathogenic mutations cause known *syndromic* genetic disorders seems a little circular. Monogenic genetic syndromes, in many ways are prime examples of pleiotropy, where a single gene affects many different and often unrelated developmental phenotypes. As such syndromic genetic disorders are pleiotropic.

*We thank the reviewer for their comments and have revised the conceptual focus toward the concept of expressivity rather than pleiotropy. This change is reflected throughout the manuscript and in the new title.*

-	I suspect that many of the differences here are related to our definition of the word “pleiotropy”. While this work demonstrates that many genetic variants in LD are demonstrating pleiotropy, pleiotropy definitions I have seen refer to a gene, rather than variants/alleles within the gene. If demonstrating novel pleiotropy is one of the intents of the manuscript, it would be more convincing if they looked at a larger number of phenotypes, those related to the syndromes and then other randomly selected phenotypes representing the other organ systems that are not known to be part of the clinical syndrome.

*We thank the reviewer for their comments. Accepting the new conceptual focus suggested by the reviewer we would point out that we tested broadly all the phenotypes identified for all the genetic syndromes, including the phenotypes without a known relation to the genetic syndrome. For example, we tested the Marfan syndrome phenotypes with all of the genetic variants included in the study. We appreciate that this point was unclear and have attempted to make this more prominent within the context of the results of the manuscript.*

*The following sentences were added in :*

- *Results (paragraph 1)*

*Here, we tested the associations between all SNPs and all phenotypes included in our study. A total of 1,824,564 test (84 phenotypes x 21,721 SNPs) was performed*

- *Methods (SNP level association section)*

*We performed the association between all 84 identified phenotypes, and all 21,721 SNPs in all the syndromic diseases genes included in our analysis.*

-	Some key question that remains unanswered in my mind, are: is the OR or Beta higher when looking at more rare SNPs? Is the OR or Beta more significant because it is in an established monogenic syndrome gene? i.e. should we be weighting variants that are known to have significant effect sizes in the rare-disease space as compared to variants that are in non-monogenic disease genes? And, importantly, might these effects be clinically significant for diagnosis? Some of these might be interesting to address in the discussion or supplemental materials.

*We thank the reviewer for these very interesting questions. We offer the following comments:*

is the OR or Beta higher when looking at more rare SNPs?

*GWAS as well as studies in mendelian diseases (PMC2914559) have demonstrated a high correlation between rare variants and high effect size. This correlation is event stronger for loss of function variants causing rare diseases or genetic syndrome as, they often have high penetrance.*

*In our study, we see a correlation between rare variants and high effect size (Pearson’s correlation -0.303, p=7.3e-22). A correlation plot between minor allele frequency and effect size may be found in the supplementary figure 7. In our study, the associations observed with rare variants were not well represented for common variants.*

Is the OR or Beta more significant because it is in an established monogenic syndrome gene?

*Malika K et al, AJHG, 2018 (PMID:30290150), in their study estimating the enrichment of mendelian disorder near GWAS regions across 62 complex traits, have showed that SNPs near phenotypically matched mendelian disorder genes have in average, a higher effect size than SNPs near protein coding genes non-phenotypically matched to mendelian disorder genes.*

*In our study, we observed in the figure 1b that the z-score for standing/sitting height ratio (one of the key phenotypes in Marfan syndrome) was higher for SNP in FBN1 (the Marfan syndrome gene) than in any other genes. Similarly, hypothyroidism often described in patient with Noonan syndrome, have the strongest association with SNPs in PTPN11.*

should we be weighting variants that are known to have significant effect sizes in the rare-disease space as compared to variants that are in non-monogenic disease genes? And, importantly, might these effects be clinically significant for diagnosis?

*To our knowledge none of the associated markers in our study were well established variants in monogenic space. For instance, none of the reported variants are clinically pathogenic from CLINVAR, loss of function variants or have a well-established biological significance. Therefore, our paper does not yet offer sufficient evidence for use in clinical prediction or decision making.*

-	The major limitation here is in the phenotype data, which the authors acknowledge in the discussion. Many of the “component” phenotypes of the syndromes are not observed in the UKBB cohort, which are primarily adults and therefore limited in pediatric phenotypes, particularly those due to dysmorphic features. Is there an independent validation cohort biobank that you could perform your analysis in, such that you could directly test these SNPs to look at specific traits (i.e TSH levels or stroke)?

*We thank the reviewer for this excellent suggestion and have validated some of the results using external summary statistics from publicly available GWAS. the following paragraph was added in the result section:*

*In order to replicate our finding, we looked in the GWAS catalogue, for sets of variants in genes in association with phenotypes or proxy-phenotypes included in our study. We excluded studies performed in non-Europeans or in the UK Biobank. We then extracted for each remaining phenotype, the association between candidate SNPs (defined SNPs significantly associated with the same phenotype in our study or in high LD (R2>0.8) with the associated SNP) and the corresponding phenotype. The association between SNPs in FBN1, PTPN11, SOS2, and JAG1 and, diastolic and systolic BP, were reported in the GWAS catalogue with the direction of effect concordant with the observed effect in our study. Similarly, the association between SNPs in PTPN11 and hypothyroidism as well as the association between JAG1 and birth weight were reported (Table S5)*

-	How were SNPs selected, i.e. are they all within the gene boundary or did you also look some distance 5’ to TSS and 3’ to the termination codon? Please add this description to your methods. Have you explored additional methods to pick the SNPs regulating genes? It might be more informative to pick SNPs that are eQTLS for the gene in question, or ones that are known to be in contact through 3D confirmation?

-	Does allele frequency seem to influence the OR or Beta value of the trait? Does it influence the p-value. Do the number of SNPs tested per gene influence performance of the gene-based SKAT test?

*We thank the reviewer for their comments. We have made more clear the criteria for selecting SNPs and allele frequencies with the following sentence for the SKAT test in the method section.*

*We performed the SKAT test on rare and common variants as well as on rare variants only. To account for the contribution of rare variants and common variants, we use SKAT CommonRare methods in which, rare and common variants are partitioned into two groups to test for the association with the phenotypes; the results of association is then combined using combined multivariate collapsing[40]. A SNP was considered rare if the Minor allele frequency was less than 0.05 (MAF<0.05). To account for their possible functional relevance, each variant was weighted in the SKAT test by their CADD score (Combined Annotation Depletion Dependent)[39,41]. Although SKAt Gene-based SKAT tests are relatively insensitive, for sensitivity analysis, we also performed SKAT using allelic frequency. Each gene was defined from 5’UTR to 3’UTR with an extra 5kb upstream, and 5kb downstream. To account for SNPs in regulatory elements of the target genes but located outside of the defined boundary, we additionally include within each target gene, SNPs in eQTL with the target gene in any tissue, but located outside of the defined boundary.*

-	Line 225 states “While classical Mendelian forms of each syndrome may be uncommon, individual phenotypes attributable to these syndromic loci appear to be common within the general population”, this is a little bit confusing, as the phenotypes are influenced by variants in the loci but they are not “attributable” to these variants. I might change the statement to read: … “individual phenotypes that *are modulated by variants within* the syndromic loci appear to be appear to be *present* within the general population.”. I think the effect you are seeing is more similar to identifying a risk locus for complex trait (individual phenotype)

*We thank the reviewer for their comments and have adjusted the text to read differently. The following sentence was used:*

*Individual phenotypes that are modulated by variants within the syndromic loci appear to be present within the general population.*

-	In the discussion (line 228), the point is made that their results support the use of a continuous phenotyping score to improve identification of rare disease using EHR. I don’t believe that referenced study seeks to identify individuals with rare syndromic mendelian disease that are using phecodes (component phenotypes) present in the EHR. I do think that that referenced paper demonstrates presence of a specific pattern component disease phenotypes might indicated presence of pathogenic Medelian genetic mutations. However, it does not really talk about how common variants in the same gene can effect matched individual phenotypes to the syndrome.

*We thank the reviewer for their comments and agree that scoring pattern of component disease phenotypes to identify “missed” individuals with mendelian disease is the focus of that particular paper. We wished to identify that paper as using a corollary approach grounded in large-scale population-based datasets even if the intent was different. We have made adjustments to the text to clarify this point.*

-	Can you corroborate other findings for significant SNPs/Loci that overlap with previous GWAS or PheWAS significant loci? I see the eQTL data mentioned in the manuscript, but well-phenotyped GWAS studies have been done on many of the anthromophoric phenotypes used here. While you may not have a causal variant in hand, maybe you have one of the causal genes that is being regulated.

*We thank the reviewer for their comments and have validated many of the findings with externally available GWAS summary statistics.*

-	Line 86 mentions that syndromic disease is due to rare and familial alleles, while the following paragraph talks about “common and rare alleles” to explore pleiotropy. Its not clear how one distinguishes rare, pathogenic vs rare and affecting individual component. How would you know that the individual does not have syndromic features—it is possible that some of the individual patients with “rare” alleles could be mildly affected (i.e. undiagnosed genetic disease?)

*We thank the reviewer for their comments. Indeed it is not possible to “diagnose” individuals with Mendelian diseases from the data available in the UK Biobank. We have added this point to the discussion of limitations.*

**Minor:**

1. How many are common, how many are rare variants that you are looking at? What is your MAF definition of rare variant vs common variant? Recent work in Wright, CF et all 2019, showed that genotyping of rare variants was notoriously inaccurate, so it would be good to know the lower limit of MAF you are considering.

*We thank the reviewer for their comments. Our methods section is now more clear about the variant inclusion criteria regarding both MAF (MAF ≤ 0.05) and imputation quality (imputation score > 0.6).*

2. For SNPS associated with Height or BMI—have they also been implicated in large scale GWAS studies?

*We thank the reviewer for their comments. Many of these variants are associated in large-scale studies of height and BMI at sub-GWAS thresholds. Where the summary statistics are available, we have integrated the replications of our findings into the manuscript.*

3. Figure 1: Please incorporate information that distinguishes Figure 1a from 1b in the legend. It is not immediately clear what the difference is, it appears that 1a is SNP-based tests that is Bonferroni corrected (and therefore corrected for multiple testing) and 1b is due to gene-based testing by SKAT.

*We thank the reviewer for their comments and have completely revised figure 1.*

4. In the main text, line130, it says “After correction for multiple testing”, and at first pass suggested that data in Figure 1A was not corrected for multiple testing. After reading several times, I realize that correction was performed, however this would benefit from simply flipping the sentence structure so you introduce gene based testing first and then mentioned that “after correction for…. Had significant hits….”

*We thank the reviewer for their comments and have corrected this phrase.*

5. Last sentence of Abstract: This sentence is a little unclear to me. I might flip the sentence to say: rare and common variants within established syndromic disease genes can have an effect on the syndrome’s individual component phenotypes within the general population.

*We thank the reviewer for their comments and have corrected this phrase.*

Reviewer #3: This is a very interesting paper describing a PheWAS based on genes important for Mendelian syndromes. I really like this idea of looking for associations in these genes with symptoms of these syndromes, whereby some alleles may be less pathogenic and thus do not lead to the full syndrome. The paper is very well written and I am very excited about this work.

That all said, I am left with some questions and wondering about some analysis choices. Also, there are perhaps some additional analyses that would really strengthen the conclusions. I will list these ideas/questions below in the order that I came across them in the paper rather than order of importance.

*We thank the reviewer for their enthusiasm and positive comments and have made revisions incorporating many of the suggestions listed below.*

1. When you did the gene burden testing in SKAT, as mentioned in abstract, is this with rare variants in those genes only? Or rare and common combined? I think this distinction is important.

*We thank the reviewer for their comments. For the SKAt analysis, we perform SKAT burden test, as well as SKAt rare only, and SKAT rare and common*

*We performed the SKAT test on rare and common variants as well as on rare variants only. To account for the contribution of rare variants and common variants, we use SKAT CommonRare methods in which, rare and common variants are partitioned into two groups to test for the association with the phenotypes; the results of association is then combined using combined multivariate collapsing[40]. A SNP was considered rare if the Minor allele frequency was less than 0.05 (MAF<0.05).*

2. In the author summary, you use the abbreviation 'SD'. This is not defined. I assume it is 'syndromic disease', but I am not certain.

*We thank the reviewer for their comments, the abbreviation SD have now been explained as Syndromic Disease (SD) in the summary*

3. I wondered why there was not a table of the gene-based results in the main paper?

*We thank the reviewer for their comments, we elected to put the gene-based results in the supplement as it was too long as to be legible or digestible in the main manuscript. However, we have now added a table (table 2) as well as an additional figure (figure 2b) for a subset of significant pairs of gene-phenotype.*

4.Minor point: RAS-opathy is spelled differently throughout. Sometimes RAS-opathie. Sometimes RAS-opathy. It should be consistent.

*We thank the reviewer for their comments, and the correct spelling has now been added as* RAS-opathy

5. In terms of the results section, several questions came to mind after reading it. Did you look at any phenotypes that are not characteristic of these syndromes? You could think of them as additional exploration or also a negative control. Did you do a formal test of pleiotropy using a method like 'pleio'? Did you do any conditional analyses to determine if the suggested pleiotropy associations are independent or whether they are present because of the correlation between the traits?

*We thank the reviewer for their comments. We performed a pleiotropy test between each SNPs and all associated phenotypes. We found significant association between all set of SNPs - associated phenotypes. The results are reported in supplementary table 7 and now discussed within the main body of the manuscript with the following paragraph:*

*To access whether association between a single SNP and multiple phenotypes are independent or due to correlation between phenotypes, we performed a formal test of pleiotropy between each independent SNP and the associated phenotypes. We found a significant association between all pleiotropic SNPS and the associated phenotypes (Table S7).*

6. I think that GTEx is misspelled throughout.

*We thank the reviewer for their comments, GTex have now been corrected to GTEx*

7. In methods, how did you bin variants for SKAT? Rare only? Rare + common? How did you define genes in terms of basepairs (up/downstream of transcription start/end?)?

*We thank the reviewer for their comments, we defined each gene as from 5’UTR to 3’UTR with an additional 50kb upstream and 50kb downstream for the SNP level association. For the gene level association, each gene was defined from 5’UTR to 3’UTR with an additional 5kb upstream and 5kb downstream. We additionally include any SNP outside the boundary that were in eQTL with the target gene in any tissue. This has been clarified in the methods section as follows:*

*Each gene was defined from 5’UTR to 3’UTR with an extra additional 5kb upstream, and 5kb downstream the gene. To account for SNPs in regulatory elements of the target gene but located outside of the defined boundary, we additionally include within each target gene, SNPs located outside of the defined boundary but in eQTL in any tissue with the target gene. For the SNP level association, we further extend the boundary of each gene to 50 kb ustream, and 50 kb downstream a gene.*

*For the SKAT analysis, we perform SKAT burden test, as well as SKAT rare only, and SKAT rare and common. The results for all SKAT association tests are present in the supplementary table 6. In addition, SKAT analysis were clarify in the methods with the following:*

*We performed the SKAT test on rare and common variants as well as on rare variants only. To account for the contribution of rare variants and common variants, we use SKAT CommonRare methods in which, rare and common variants are partitioned into two groups to test for the association with the phenotypes; the results of association is then combined using combined multivariate collapsing[40]. A SNP was considered rare if the Minor allele frequency was less or equal to 0.05 (MAF ≤0.05). To account for their possible functional relevance, each variant was weighted in the SKAT test by their CADD score (Combined Annotation Depletion Dependent)[39,41].*

8. Did you also look at burden tests like regression or wilcoxon? It is known that SKAT can have a high type I error rate, so it would be a good idea to be conservative with those findings.

*We thank the reviewer for their comments. We did not perform wilcoxon testing as the burden testing implemented in SKAT permits simultaneous analysis of variants with opposite directions, conferring both positive and negative effects upon a single outcome. As we did not have a priori hypotheses about the direction of effects for individual variants (increase or decrease in height, or increase or decrease in the risk of a binary outcome), we elected not to perform wilcoxon based burden tests.*

9. Much like GWAS, analyses like these could be prone to false positives. It is possible to look for replication of these signals either through independent datasets or even cross-validation within this large dataset.

*We thank the reviewer for their comments, agree with their skepticism, and have attempted to replicate the signals where possible using publicly available summary statistics from GWAS data. The following were clarify in the results section of manuscript:*

*In order to replicate our finding, we looked in the GWAS catalogue, for sets of variants in genes in association with phenotypes or proxy-phenotypes included in our study. We excluded studies performed in non-Europeans or in the UK Biobank. We then extracted for each remaining phenotype, the association between candidate SNPs (defined SNPs significantly associated with the same phenotype in our study or in high LD (R2>0.8) with the associated SNP) and the corresponding phenotype. The association between SNPs in FBN1, PTPN11, SOS2, and JAG1 and, diastolic and systolic BP, were reported in the GWAS catalogue with the direction of effect concordant with the observed effect in our study. Similarly, the association between SNPs in PTPN11 and hypothyroidism as well as the association between JAG1 and birth weight were reported (Table S5)*

10. Finally, did you consider doing a test of epistasis in these analyses? Since there is a lot of evidence of epistasis in the different symptoms of Mendelian disease, and epistasis and pleiotropy often co-occur, it would be very cool to look to see if there is any evidence for nonlinear interactions between these genes, or variants in these genes.

*We thank the reviewer for their comments and have performed a test of epistasis, defining epistasis as a statistically significant interaction term between two independent SNPs. These results are now reported and integrated into the paper in supplementary table 8. Additionally these results were reported as follows:*

*To evaluate whether associations between a phenotype and multiple SNPs were explained by variant-variant interaction or epistasis we performed a stepwise linear or logistic regression with interaction terms between pairs of associated SNPs for each phenotype. After multiple testing correction, none of the SNP pairs displayed significant interactions with each other (Table S8).*

Reviewer #1: No: Data from UKBB cannot be provided and so I do not believe the authors can do so. They do not provide code/scripts that were used, which should be possible.

Reviewer #2: Yes

Reviewer #3: Yes
