# Supplementary material for: A phenome-wide association study of 26 mendelian genes reveals phenotypic expressivity of common and rare variants within the general population
Source: PLoS Genet. 2020 Nov 23;16(11):e1008802. doi: 10.1371/journal.pgen.1008802 (PMC7735621; doi:10.1371/journal.pgen.1008802)
Supplement: Attachment — Submitted filename: response_letter_rev2_CT.docx [file pgen.1008802.s019.docx]

Reviewer #1: The authors have responded well to all of my comments but one.
In my original review, I stated that

They do not provide code/scripts that were used, which should be possible.

=> The authors seem to have ignored this comment. They should share the scripts they used for analysis and provide sufficient documentation such that others can reproduce their results. Of course they cannot share the UKBB data but new users can be expected to download this themselves.

*We thank the reviewer for this comment and have shared the scripts for analysis at* <https://github.com/cathynes/PHEWAS-SD>

Reviewer #2: This is a revision that I think has come back improved and more clearly written. This work, which looks at component phenotypes associated with 26 Mendelian disease genes using UK Biobank. It is quite exciting to think about how Mendelian disease genes can be used to interpret common variation that regulates those genes and component phenotypes to a lesser degree. Overall, I am excited by the work which starts to consider how we can unify our knowledge bases between Mendelian syndromes and complex trait genetics to improve our understanding of genetic-influence on human traits.

*We thank the reviewer for this comment and appreciate their interest in our work.*

I don’t have major comments and minor comments are below

Minor:
line 178: should use “weighted” not weight
Figure 2: first line: should say “Plot of PheWAS results for all genes and all phenotypes”
Line 395: The last sentence of the manuscript is a little unclear and would benefit from some simplification to get their message across

*We thank the reviewer for these comments and have made the appropriate changes.*

Reviewer #3: The authors did a very nice job revising the manuscript based on the reviewer comments. One remaining issue that I see is that they changed the title, which I think was the right thing to do, however I think they have a typo. It now says four syndromic genes. In this study, they did a PheWAS for more than 4 genes. They used gene sets from four syndromic diseases. I think they meant four diseases.

*We thank the reviewer for these comments and have made a clarification to the title which now reads as,*

*“*A phenome-wide association study of 26 Mendelian diseases genes targeting four genetic syndromes, reveals phenotypic expressivity of common and rare variants within the general population*.”*

Also in the discussion, the authors mention 4 syndromic loci. The explored 26 genes from the 4 syndromes. So it is unclear to me why they keep saying 4 syndromic loci.

*We thank the reviewer for making this point and note that in the parlance of clinical genetics, the term “locus” may be used to refer to a band or sub-band physical region of a chromosome. A locus such as 22q11.2 may contain multiple genes as in DiGeorge syndrome. Alternatively, the term “locus” can be used interchangeably with the term “gene”. In our usage in this paper we have combined these two meanings, perhaps to the confusion of the reader. We have made clarifications throughout. For example, in the lines 344 and 352, Instead “variants at syndromic loci”, we used term “variants within loci of syndromic disease genes”.*

Finally, the authors should be careful about how they refer to SNPs. Sometimes they use "SNPs" and other times "SNPS".

*We thank the reviewer for noticing the inconsistency and have corrected all to “SNPs”.*

**Have all data underlying the figures and results presented in the manuscript been provided?**
Large-scale datasets should be made available via a public repository as described in the *PLOS Genetics* [data availability policy](http://journals.plos.org/plosgenetics/s/data-availability), and numerical data that underlies graphs or summary statistics should be provided in spreadsheet form as supporting information.

Reviewer #1: No: It is not possible to share the UKBB data

Reviewer #2: Yes

Reviewer #3: Yes

PLOS authors have the option to publish the peer review history of their article ([what does this mean?](https://journals.plos.org/plosgenetics/s/editorial-and-peer-review-process#loc-peer-review-history)). If published, this will include your full peer review and any attached files.


If you choose “no”, your identity will remain anonymous but your review may still be made public.


**Do you want your identity to be public for this peer review?** For information about this choice, including consent withdrawal, please see our [Privacy Policy](https://www.plos.org/privacy-policy).

Reviewer #1: No

Reviewer #2: No

Reviewer #3: No
